# Lipid profile and response to statin therapy in patients with hypopituitarism

**DOI:** 10.20945/2359-3997000000292

**Published:** 2020-10-09

**Authors:** Graziela Rissetti, Débora Zeni, Bárbara Roberta Ongaratti, Júlia Fernanda Semmelmann Pereira-Lima, Carolina Garcia Soares Leães Rech, Miriam da Costa Oliveira

**Affiliations:** 1 Universidade Federal de Ciências da Saúde de Porto Alegre Programa de Pós-Graduação em Patologia Porto Alegre RS Brasil Programa de Pós-Graduação em Patologia, Universidade Federal de Ciências da Saúde de Porto Alegre (UFCSPA), Porto Alegre, RS, Brasil; 2 Universidade Federal de Ciências da Saúde de Porto Alegre Complexo Hospitalar Santa Casa Centro de Neuroendocrinologia Porto Alegre RS Brasil Centro de Neuroendocrinologia, Complexo Hospitalar Santa Casa, Universidade Federal de Ciências da Saúde de Porto Alegre (UFCSPA), Porto Alegre, RS, Brasil

**Keywords:** Hypopituitarism, pituitary adenoma, lipid profile, statin response, simvastatin

## Abstract

**Objective:**

Dyslipidemia is prevalent among patients with hypopituitarism, especially in those with growth hormone (GH) deficiency. This study aimed to evaluate the response to statin therapy among adult patients with dyslipidemia and hypopituitarism.

**Subjects and methods:**

A total of 113 patients with hypopituitarism following up at a neuroendocrinology unit were evaluated for serum lipid levels. Dyslipidemia was diagnosed in 72 (63.7%) of these patients. A control group included 57 patients with dyslipidemia and normal pituitary function. The distribution of gender, age, weight, and dyslipidemia type was well balanced across both groups, and all participants were treated with simvastatin at doses adjusted to obtain normal lipid levels.

**Results:**

Patients with hypopituitarism and dyslipidemia presented deficiency of TSH (69%), gonadotropins (69%), ACTH (64%), and GH (55%) and had a similar number of deficient pituitary axes compared with patients with hypopituitarism but without dyslipidemia. All patients with dyslipidemia (with and without hypopituitarism) had lipid levels well controlled with doses of simvastatin ranging from 20-40 mg/day. The mean daily dose of simvastatin was not significantly different between patients with and without hypopituitarism (26.7 versus 23.5 mg, p = 0.10). Similarly, no significant variation in simvastatin dose was observed between patients with different causes of hypopituitarism, presence or absence of GH deficiency, number of deficient pituitary axes, prior pituitary radiation therapy or not, and presence or absence of obesity.

**Conclusions:**

Patients with GH deficiency without GH replacement showed good response to simvastatin at a mean dose equivalent to that used in individuals with dyslipidemia and normal pituitary function.

## INTRODUCTION

Hypopituitarism is a rare condition with an incidence of 2.07 to 4.21 new cases per hundred thousand inhabitants per year ( 1 , 2 ). It can be caused by hypothalamic or pituitary diseases, especially pituitary adenomas ( 3 ). Hypopituitarism is associated with increased morbidity and mortality, usually secondary to cardiovascular and cerebrovascular diseases in parallel to the presence of metabolic syndrome, changes in body composition, insulin resistance, systemic arterial hypertension, atherosclerosis, and/or unfavorable lipid profile characterized by increased levels of total cholesterol, low-density lipoprotein (LDL) cholesterol, and triglycerides ( 4 - 7 ). Growth hormone (GH) deficiency is an additional independent risk factor for the occurrence of dyslipidemia ( 8 - 10 ).

Levels of total cholesterol, LDL cholesterol, and triglycerides are elevated in individuals with hypopituitarism compared with controls ( 11 ). However, data about the prevalence of dyslipidemia in patients with hypopituitarism are scarce. Patients with hypopituitarism in Japan have an elevated prevalence of dyslipidemia – up to 30% and 40% among men and women, respectively – although these results cannot be generalized to other populations ( 12 ). Still, no data are available in the literature regarding the statin dose required to normalize lipid levels in patients with hypopituitarism. This information is important, especially considering the challenges that adults with GH deficiency in some countries face to obtain recombinant human GH from the public health care system.

Based on these considerations, this study aimed to evaluate the response of serum lipid levels to simvastatin among patients with dyslipidemia and hypopituitarism without GH deficiency or with GH deficiency but without GH replacement, and to compare the response of these patients with that from individuals with normal pituitary function.

## SUBJECTS AND METHODS

This prospective cohort study was conducted at a neuroendocrinology unit in southern Brazil. The study was approved by the Research Ethics Committee of *Complexo Hospitalar Santa Casa* at *Universidade Federal de Ciências da Saúde de Porto Alegre* (CAAE number 15459713.3.0000.5335) and was conducted in compliance with the Declaration of Helsinki. All patients were informed about the study objectives and signed a free and informed consent form.

The study included patients diagnosed with hypopituitarism (defined by deficiency of one or more anterior pituitary hormones) and dyslipidemia treated with simvastatin. Hypopituitarism was confirmed based on the following findings for each pituitary hormone deficiency: (i) hypothyroidism – low serum free T4 (FT4) level in the presence of normal or slightly increased TSH level; (ii) hypocortisolism – serum cortisol level below the reference range or the expected value post-stimulation with ACTH; (iii) hypogonadism – history compatible with hypogonadism (during childbearing age) or low levels of gonadotropins (during menopause) in women, or low gonadotropin levels in the presence of low total testosterone in men; and (iv) GH deficiency – serum IGF-1 levels below the reference value for sex and age.

All patients with hypopituitarism were receiving adequate and stable doses of hormone replacement therapy for each deficient pituitary axis – except for GH replacement – for at least 6 months. Hormone replacement comprised levothyroxine at doses adjusted by FT4 levels in patients with hypothyroidism; prednisone in usual replacement doses in those with cortisol deficiency; and a combination of estrogen/progestin (women of reproductive age) or injectable testosterone ester (men) in those with hypogonadism.

In the study period, 113 patients with hypopituitarism were seen at the neuroendocrinology unit, of which 72 had dyslipidemia (63.7%). These patients presented with isolated hypercholesterolemia (LDL cholesterol > 160 mg/dL), isolated hypertriglyceridemia (triglycerides > 150 mg/dL), mixed hyperlipidemia with increased LDL cholesterol levels (LDL cholesterol > 160 mg/dL and triglycerides > 150 mg/dL), or isolated decreased HDL cholesterol or decreased HDL cholesterol in combination with increased LDL cholesterol or triglycerides (HDL cholesterol < 40 mg/dL in men and < 50 mg/dL in women) ( 12 , 13 ).

**Figure 1:**
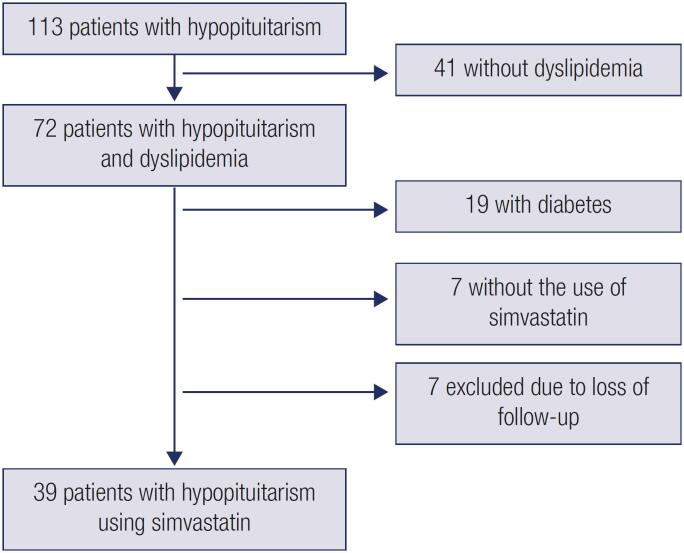
Study flow chart.

Patients with diabetes mellitus were excluded from the group with dyslipidemia since diabetes may lead to dyslipidemia and could be a source of bias. After excluding patients who were lost to follow-up and those with type 2 diabetes mellitus or using other hypolipidemic drugs, the final sample comprised 39 patients with hypopituitarism using simvastatin alone ( Figure 1 ). Table 1 shows the profile of the participants in the overall cohort.

**Table 1:** General characteristics of patients with hypopituitarism and controls

		Patients with hypopituitarism, n = 113 (n,%)	Patients without hypopituitarism, n = 57 (n,%)	Patients with hypopituitarism and using simvastatin, n = 39 (n,%)
Gender				
	Female	59 (52.2)	31 (54.4)	21 (53.8)
	Male	54 (47.8)	26 (45.6)	18 (46.2)
Mean age (years)		49 (16-87)	53 (20-86)	52 (18-87)
Nutritional status *				
	Underweight	3 (2.7)	2 (3.5)	1 (2.6)
	Eutrophic	17 (15)	14 (24.6)	6 (15.3)
	Overweight	37 (32.8)	15 (26.3)	13 (33.3)
	Obese	56 (49.5)	26 (45.6)	19 (48.7)
Dyslipidemia		72 (63.7)	57	39
Type 2 diabetes mellitus		25 (22.1)	0	0

* Nutritional status was assessed according to body mass index (BMI), calculated as current weight (kg)/height (m^2^). The patients were classified according to BMI as underweight (<18.5 kg/m^2^), eutrophic (18.5-24.9 kg/m^2^), overweight (25.0-29.9 kg/m^2^), or obese (≥30.0 kg/m^2^).

The control group comprised 57 patients following up at the same hospital and recruited from another outpatient clinic. Participants in this group had normal thyroid function, a previous diagnosis of dyslipidemia with ongoing treatment with simvastatin, and no history of pituitary dysfunction or diabetes. In this group, the participants had a mean age of 53 years (range 20-86 years), 54% were women, and 46% were obese ( Table 1 ).

The initial evaluation of the patients included the measurement of serum lipid levels and characterization of the dyslipidemia. In the control group, the information on the type of dyslipidemia was retrieved from medical records. We collected data about other comorbidities, current medications along with dose and duration of use, and adherence to treatment from the participants in both groups. In the group of patients with hypopituitarism, we also collected information about disease duration and results of laboratory tests relevant in the follow-up of pituitary hormone replacement. Follow-up assessments included simvastatin-related and lipid profile data, along with a routine evaluation of the patients with hypopituitarism.

Simvastatin was administered at night and adjusted in both groups every 8-12 weeks to achieve lipid control goals. The patients were then followed up for a minimum of 6 months.

The data collected were analyzed using the software Statistical Package for the Social Sciences, version 22.0 (SPSS, IBM Corp. Armonk, NY, USA). Quantitative data were described by mean values. Asymmetry was considered to be present when the standard deviation was greater than half of the mean value. We used the chi-square test for comparisons between categorical variables, or the Kruskal-Wallis test when these variables were asymmetric. The adopted level of statistical significance was 5%.

## RESULTS

Hypopituitarism was associated with pituitary adenomas in 63% of the patients, who were treated with surgery (69%) and/or radiotherapy (22%). The frequencies of TSH, ACTH, gonadotropins, and GH deficiency in patients with hypopituitarism using simvastatin were, respectively, 82%, 77%, 74%, and 52%.

In the group of patients with hypopituitarism and dyslipidemia, the mean age was 49.0 ± 15.9 years (range 16-87 years), 59 (52%) were female, 56 (50%) were obese, and 37 (33%) were overweight ( Table 1 ).

Among patients with hypopituitarism, the frequency of dyslipidemia was not significantly different between those with and without prior radiotherapy (72% versus 61%, respectively, *p* = 0.35) and between those with and without obesity (69% versus 58%, respectively, *p* = 0.24). The combination of types of pituitary hormone deficiencies in the study population was comparable to that in patients with hypopituitarism in the general population. The types and number of affected hormonal axes were not significantly different among patients with hypopituitarism with versus without dyslipidemia.

The distribution of the types of dyslipidemia was similar in the groups with and without hypopituitarism ( Table 2 ).

**Table 2:** Distribution of dyslipidemia types^12^ across groups in the study cohort *

	Patients with hypopituitarism, n = 39 (%)	Patients without hypopituitarism, n = 57 (%)
HChol	8 (20)	20 (35)
HTG	9 (23)	6 (10)
HDL-C mixed	14 (36)	15 (26)
HDL-Chol	8 (20)	16 (28)

HChol, isolated hypercholesterolemia; HTG, isolated hypertriglyceridemia; HDL-C mixed, mixed hyperlipidemia with increased LDL cholesterol levels; HDL-Chol, isolated decreased HDL cholesterol or decreased HDL cholesterol in combination with increased LDL cholesterol or triglycerides.

* No significant difference between groups. Chi-square test, *p* = 0.15.

Pretreatment levels of total cholesterol, HDL cholesterol, LDL cholesterol, and triglycerides among patients with dyslipidemia and hypopituitarism were, respectively, 199-342 mg/dL (254 ± 35 mg/dL), 38-91 mg/dL (57 ± 16 mg/dL), 97-226 mg/dL (159 ± 32.4 mg/dL), and 110-364 mg/dL (214 ± 87 mg/dL). The median LDL cholesterol level after treatment was higher in individuals with GH deficiency, but no significant differences were observed when the median levels of all lipids were compared between patients with hypopituitarism versus controls ( Table 3 ).

**Table 3:** Lipid levels after treatment

	Patients with hypopituitarism		Patients without hypopituitarism
	With GH deficiency	Without GH deficiency	
Cholesterol (mg/dL)	197 (167-211)	171 (152-192)	183 (169-193)
HDL cholesterol (mg/dL)	47 (40-52)	51 (45-61)	46 (39-56)
LDL cholesterol (mg/dL)	119 (97-141) *	91 (81-113) *	112 (96-121)
Triglycerides (mg/dL)	131 (89-173)	128 (107-206)	126 (89-143)

The data are presented as median (25-75% interquartile range).

* Kruskal-Wallis, *p* = 0.005.

In both groups with and without hypopituitarism, lipid levels were controlled with doses of simvastatin ranging from 20 to 40 mg/day (mean dose 25.1 mg/day). The mean simvastatin dose was 26.7 mg in patients with hypopituitarism and 23.5 mg in those with normal pituitary function and was not significantly different between both these groups ( *p* = 0.10).

There was no significant difference in the mean daily dose of statin among patients with hypopituitarism of different etiologies (25 mg for those with adenomas versus 28 mg for those with hypopituitarism due to other causes, *p* = 0.27), patients with untreated GH deficiency versus those with no GH deficiency (21.7 mg versus 29.1 mg, respectively, *p* = 0.054), patients with prior radiotherapy compared with those without prior radiotherapy (23.1 mg versus 28.5 mg, respectively, *p* = 0.07), and individuals with obesity versus those without obesity (25.6 mg versus 24.2 mg, respectively, *p* = 0.46).

A separate analysis of the group with hypopituitarism and simvastatin use showed no significant difference in mean simvastatin dose among patients with different numbers of deficient pituitary axes ( *p* = 0.89) ( Table 4 ).

**Table 4:** Mean dose of simvastatin relative to the number of pituitary axes affected in patients with hypopituitarism *

	Simvastatin, mean dose (mg)
1 deficient axis	26.7
2 deficient axes	26.2
3 deficient axes	27.8
4 deficient axes	24.0

* No significant differences were observed between groups. Chi-square test, *p* = 0.89.

## DISCUSSION

The proportion between men and women affected by hypopituitarism in the present study is similar to that reported in different studies ( 6 , 7 , 14 , 15 ), although in some series, males are slightly more frequently affected by the disease, reaching up to 62% in a study by Bülow and cols. (1997) ( 16 , 17 ). The mean age of 49 years found in our study is also aligned with data available in the literature, in which the mean age of individuals with hypopituitarism ranges from 43 to 59 years ( 6 , 7 , 14 - 17 ).

Some researchers have shown that the prevalence of overweight and obesity is elevated in patients with hypopituitarism, with variations ranging from 10% in a study by Murakami and Kato (2003), who defined obesity by body mass index ( 12 ), to 53% in a study by Joustra and cols. (2014), who defined obesity by waist circumference ( 18 ). Other studies have found no increased prevalence of obesity in hypopituitarism ( 5 ). The present study used body mass index greater than 30 kg/m^2^ to characterize obesity and found a high prevalence of this condition (50% of the sample). However, the prevalence of obesity in our study was not different between patients with hypopituitarism and control subjects with dyslipidemia.

Studies evaluating glucose tolerance and/or insulin resistance in patients with hypopituitarism have shown a prevalence of glycemic alterations of about 30% ( 5 , 18 ). Diabetes mellitus was present in 22% of our sample, which is consistent with data from the literature.

According to Murakami and Kato (2003), dyslipidemia affects approximately 36% of the patients with hypopituitarism ( 12 ), a percentage lower than the one found in the present study (64%). This difference can be explained by the different cutoff values for lipid levels adopted by each study for the diagnosis of dyslipidemia. There are no data available on the distribution of dyslipidemia in patients with hypopituitarism, which in the present study was found to be similar when patients with and without hypopituitarism were compared.

Among patients with hypopituitarism in our study, no significant differences were observed between the type and number of deficient pituitary axes in patients with dyslipidemia versus those without dyslipidemia. This result draws attention since Kaji and cols. (2007) have shown associations between deficiency of different pituitary hormones and increased prevalence of dyslipidemia, particularly between treated TSH deficiency and untreated GH deficiency, and between treated TSH and gonadotropin deficiencies ( 19 ).

Studies on the use of statin in patients with dyslipidemia and hypopituitarism are rare. The specific choice for simvastatin in our study was based on the extensive use of this drug to treat dyslipidemia. This is also a low-cost medication of easy access to the general population since it is distributed free of charge by the Brazilian Unified Healthcare System.

Several studies have addressed the relationship between GH deficiency and dyslipidemia in hypopituitarism, showing conflicting results. Some authors have observed an association between untreated GH deficiency and increased risk of cardiovascular disease secondary to a higher frequency of metabolic syndrome and its components ( *i.e.,* central adiposity, insulin resistance, decreased levels of HDL cholesterol, and increased levels of total cholesterol, LDL cholesterol, and triglycerides) ( 8 , 9 ). For some authors, GH replacement is fundamental in the treatment of patients with dyslipidemia and hypopituitarism. Verhelst and Abs (2009) have shown that treatment with GH can improve lipid profile and obesity in patients with GH deficiency ( 10 ). Additionally, GH replacement has been associated with a slight increase in insulin sensitivity ( 9 ). Kaji and cols. (2007) have shown that the prevalence of cardiovascular risk factors is not significantly different between patients with untreated GH deficiency versus those with normal GH secretion ( 19 ). On the other hand, Monson and cols. (2007) have shown that GH replacement can bring additional benefits in reducing cardiovascular and cerebrovascular risk among patients with dyslipidemia receiving lipid-lowering therapy with statin ( 20 ). In our study, even in the absence of GH replacement, we obtained good results in controlling the lipid profile of patients with hypopituitarism using similar simvastatin doses as those used by patients with dyslipidemia but without hypopituitarism.

Some studies have shown that patients undergoing cranial radiotherapy have a higher risk of metabolic syndrome ( 6 , 15 , 21 , 22 ), but no studies in the literature have associated radiotherapy of the sellar region with a higher incidence of lipid abnormalities. The present study found that exposure to radiation did not increase the frequency of dyslipidemia or interfered with the mean dose of simvastatin required to control lipid levels.

Several studies have shown an increased prevalence of dyslipidemia in patients with obesity, and that obesity *per se* is a secondary cause of dyslipidemia ( 23 , 24 ). However, the presence of obesity in our study was not associated with an increased incidence of dyslipidemia or with a significant difference in the mean dose of simvastatin needed to control lipid levels in patients with hypopituitarism.

Concerning the dose of simvastatin required to normalize the lipid profile in the present study, the low dose required to normalize the levels of total cholesterol was noteworthy and probably explained by the modestly increased baseline levels (mean 253 mg/dL). We observed no difference in the dose of simvastatin used by patients (i) with versus without hypopituitarism, (ii) with hypopituitarism related to pituitary adenomas versus hypopituitarism not related to pituitary adenomas, (iii) without GH deficiency versus with GH deficiency without replacement, (iv) with versus without prior history of radiotherapy, and (v) with versus without obesity.

Limitations of this study include the number of individuals in the cohort and the presence of confounding factors that may have affected the lipid profile of the subjects such as familial hyperlipidemia, diet, and exercise, which the study was not designed to address. Another point to be investigated in the future is the possibility of a different percentage of reduction in lipid levels in groups with and without hypopituitarism. However, the results allow us to conclude that dyslipidemia affects 64% of the patients with hypopituitarism and suggest that these individuals, regardless of the presence or absence of GH deficiency, respond adequately to treatment with simvastatin at a mean dose equivalent to that used by individuals with dyslipidemia and normal pituitary function. These data are important in the follow-up of patients with hypopituitarism, especially among those without access to GH replacement.
